# Identification and characterization of the intracellular poly-3-hydroxybutyrate depolymerase enzyme PhaZ of Sinorhizobium meliloti

**DOI:** 10.1186/1471-2180-10-92

**Published:** 2010-03-27

**Authors:** Maria A Trainer, David Capstick, Alicja Zachertowska, Kathy N Lam, Scott RD Clark, Trevor C Charles

**Affiliations:** 1 Department of Biology, University of Waterloo, 200 University Ave W, Waterloo, ON N2L 3G1, Canada; 2 Current address: Council of Canadian Academies, 180 Elgin St, Suite 1401, Ottawa, ON, K2P 2K3, Canada; 3 Current address: Department of Biology, McMaster University, 1280 Main St West, Hamilton, ON, L8S 4K1, Canada; 4 Current address: Department of Molecular Genetics, University of Toronto, 1 King's College Circle, Toronto, ON, M5S 1A8, Canada

## Abstract

**Background:**

*S. meliloti *forms indeterminate nodules on the roots of its host plant alfalfa (*Medicago sativa*). Bacteroids of indeterminate nodules are terminally differentiated and, unlike their non-terminally differentiated counterparts in determinate nodules, do not accumulate large quantities of Poly-3-hydroxybutyrate (PHB) during symbiosis. PhaZ is in intracellular PHB depolymerase; it represents the first enzyme in the degradative arm of the PHB cycle in *S. meliloti *and is the only enzyme in this half of the PHB cycle that remains uncharacterized.

**Results:**

The *S. meliloti phaZ *gene was identified by *in silico *analysis, the ORF was cloned, and a *S. meliloti phaZ *mutant was constructed. This mutant exhibited increased PHB accumulation during free-living growth, even when grown under non-PHB-inducing conditions. The *phaZ *mutant demonstrated no reduction in symbiotic capacity; interestingly, analysis of the bacteroids showed that this mutant also accumulated PHB during symbiosis. This mutant also exhibited a decreased capacity to tolerate long-term carbon starvation, comparable to that of other PHB cycle mutants. In contrast to other PHB cycle mutants, the *S. meliloti phaZ *mutant did not exhibit any decrease in rhizosphere competitiveness; however, this mutant did exhibit a significant increase in succinoglycan biosynthesis.

**Conclusions:**

*S. meliloti *bacteroids retain the capacity to synthesize PHB during symbiosis; interestingly, accumulation does not occur at the expense of symbiotic performance. *phaZ *mutants are not compromised in their capacity to compete for nodulation in the rhizosphere, perhaps due to increased succinoglycan production resulting from upregulation of the succinoglycan biosynthetic pathway. The reduced survival capacity of free-living cells unable to access their accumulated stores of PHB suggests that PHB is a crucial metabolite under adverse conditions.

## Background

Several genera of soil bacteria can enter into nitrogen-fixing symbioses with leguminous plants. These genera, commonly referred to as the 'rhizobia', include *Sinorhizobium*, *Rhizobium*, *Bradyrhizobium*, and *Azorhizobium*. Formation of specialized, microaerophilic nodules on the roots of the host plant are elicited by the bacteria. Following infection and colonization of the nodule tissue, the bacteria undergo differentiation into a mature state known as the bacteroid, which can reduce atmospheric dinitrogen to ammonia. Bacteroid metabolism is dominated by the production of fixed nitrogen, which is transferred directly to the host plant. This energetically expensive reaction, catalyzed by the nitrogenase complex, is fuelled by the host plant by provision of fixed carbon, generally in the form of C4-dicarboxylates such as malate and succinate [1-3]. Most of the carbon supplied by the plant is used to fuel nitrogen fixation, however, under certain circumstances, some of the carbon appears to be diverted by the bacteroid into the production of intracellular carbon storage polymers such as poly-3-hydroxybutyrate (PHB). This is a characteristic of bacteroids found in determinate nodules but not of indeterminate nodules (reviewed in [4]). Within the bacteroid, PHB deposits can be visualized as defined, electron-transparent granules located within the cytoplasm [5-7].

*S. meliloti *forms indeterminate nodules on the roots of its host plant alfalfa (*Medicago sativa*). These nodules are characterized by the existence of a persistent apical meristem and an elongated morphology. Within the nodule, the bacteroids persist and progress through defined zones of bacteroid differentiation [8]. Indeed, loss of PHB granules from the cytoplasm of the bacteria invading indeterminate nodules is a well-documented phenomenon that occurs at a specific point within bacteroid development [9].

Bacteroids of indeterminate nodules undergo such large physiological and metabolic changes relative to those of determinate nodules [10] that, until recently, it was unclear whether mature bacteroids within indeterminate nodules retained the capacity to synthesize and store PHB. A recent study [11] clearly demonstrated that bacteroids of *R. leguminosarum *bv. *viciae*, which forms indeterminate nodules on pea plants, retain the capacity to synthesize and store large quantities of PHB but only when carbon supply is in excess and bacteroid metabolism is limited by the availability of a key nutrient (reviewed in [4]). During saprophytic growth, PHB accumulation occurs during periods of nutrient deprivation when carbon is in excess. This strategy is employed by many species of bacteria. The first step in PHB degradation is catalyzed by a substrate-specific depolymerase. PHB undergoes a transition from an amorphous granule in the intracellular state to a denatured semi-crystalline form upon release into the environment. As a result, different PHB depolymerases are employed depending on the nature of the substrate. While extracellular depolymerases have been identified and characterized in a wide variety of bacteria, very little is yet known about their intracellular counterparts. To date, only a handful of intracellular PHB depolymerases have been reported in the literature, most of which appear to lack the typical lipase box motif (Gly-X-Ser-X-Gly) associated with extracellular PHB depolymerases [12-17]. While the enzymes responsible for the synthesis and storage of PHB have been characterized in a wide variety of bacteria, including the rhizobia (reviewed in [4]), only a few studies have investigated the role of intracellular PHB depolymerases and, to date, no studies have reported the characterization of a rhizobial PHB depolymerase.

Here we report the cloning and characterization of PhaZ from *S. meliloti *after its identification as the putative intracellular PHB depolymerase based on *in silico *analyses of the genome sequence and comparisons to other intracellular PHB depolymerase sequences. This work is the first report of a PHB depolymerase mutant in *S. meliloti *and, indeed, in the rhizobia. This work also represents the final step in genetic characterization of the complete PHB cycle in these bacteria, as all other enzymes of both the synthetic and degradative pathways have been previously studied [3,5,6,8,18,19]. To the best of our knowledge, this work also documents the first confirmed example of the presence of intracellular PHB granules in N_2_-fixing bacteroids of *S. meliloti*.

## Results and Discussion

### Identification of the S. meliloti phaZ Open Reading Frame and Construction of an S. meliloti phaZ mutant

The *phaZ *gene was identified as a 1272 bp open reading frame SMc02770 in the *S. meliloti *genome sequence [20] by comparison to *phaZ *of *Cupriavidus necator *[13]. The amino acid sequences of these two proteins share 51% identity. Interestingly, like *phaZ *of *C. necator*, the PhaZ protein of *S. meliloti *does not possess a Gly-X-Ser-X-Gly lipase box motif [21] that is characteristic of many extracellular PHB depolymerases. The absence of this motif implies that these intracellular PhaZ homologues may use a different active site structure to extracellular PHB depolymerases. Primers were designed to internal regions of *phaZ *to amplify a fragment (from S35 to F292) by PCR, and the resultant 835 bp fragment was cloned into pGEM^®^-T Easy (Promega) to generate pAZ101. An internal disruption of the cloned *phaZ *fragment was generated by introducing a ΩSmSp cassette as a Cfr91 fragment into the unique KpnI site at 299 bp to yield pAZ102. The *phaZ*::ΩSmSp was subsequently excised as an EcoRI fragment and subcloned into pK19*mobsacB *to give pAZ103. pAZ103 was introduced into *S. meliloti *Rm5000 by triparental mating using *E. coli *MT616 as a helper strain. Single recombinants were identified by selecting for Rf^*R*^, Sm^*R*^, Sp^*R *^transconjugants. Putative double recombinants were identified by plating onto TY Sm Sp Sucrose (5%). Subsequent screening for loss of vector-encoded Nm^*R *^confirmed the loss of pK19*mobsacB*. The resultant Rf^*R*^, Sm^*R*^, Sp^*R*^, Nm^*S*^*phaZ *mutant was designated Rm11417. The mutagenesis was confirmed by Southern blot using the *phaZ *PCR product as a probe. The probe hybridized to a 1.55 kb EcoRI fragment of genomic DNA in the wild-type strain Rm5000, and to a 3.55 kb fragment in Rm11417, confirming the presence of the 2 kb ΩSmSp cassette (data not shown). This mutation was transduced into Rm1021 using the *ϕ*M12 phage by standard techniques [22] and the resultant mutant was designated Rm11430.

### Cloning of phaZ gene for complementation assays

Primers Smc02770F 5'-CCTAAGCTTATGTTCTACCAGCTTTACGAGATGAAC-3' and Smc02770R 5'-CGAAAGCTTTTAGTGATGGTGATGGTGATGGGCCGACTTGCCGCCCTTG-3' were designed to the 5' and 3' regions of SMc02770, incorporating HindIII sites into the 5' and 3' ends as well as a 3' terminal His tag. The PCR product was cloned as a HindIII fragment into pRK7813 and the resultant construct was named pMA157. This construct was introduced into Rm11430 by triparental conjugation using MT616 as the mobilizer strain.

### Growth Phenotype of Rm11430 and ability to survive long-term carbon starvation

Mutants of *phaC*, *phaB*, and *bdhA *all demonstrate impaired growth on PHB cycle intermediates [23,24]. To determine if a lesion in *phaZ *resulted in a similar impairment in the capacity of *S. meliloti *to utilize PHB Cycle intermediates, the growth of Rm11430 was compared to that of Rm1021, Rm11105 [23], Rm11107 [23] and Rm11347 [24] on TY, YMA, and minimal media containing either 15 mM acetate (A), acetoacetate (AA) or D-3-hydroxybutyrate (HB) as sole carbon sources. No difference in growth phenotype was observed between Rm11430 and Rm1021 (Table 1).

**Table 1:** Growth Phenotypes of *S. meliloti *PHB Cycle Mutants

Strain	Relevant Characteristics	YMA Phenotype	Carbon Source Utilization			
			
			Glucose	D-3-HB	Acetate	AA
Rm1021	wild-type	Mucoid	+	+	+	+
Rm11105	*phaC*::Tn*5*	Dry	+	-	+	-
Rm11107	*bdhA*::Tn*5*	Mucoid	+	-	+	+
Rm11347	*phaB*Ω	Dry	+	-	+	-
Rm11430	*phaZ*ΩSmSp	Mucoid	+	+	+	+

**Figure 1:**
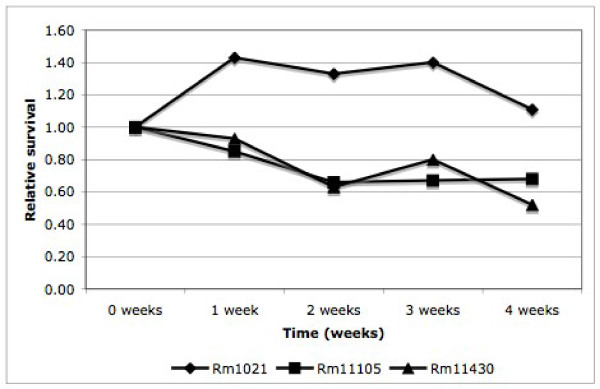
**Viable cell counts of S. meliloti PHB mutants following incubation in minimal media with no exogenous carbon source added**. Values presented are the average of three independent cultures. Rm1021 cells are able to maintain viability for almost 4 weeks following transition to a carbon-free environment. In contrast, both Rm11105 and Rm11430 demonstrate a significant decrease in viability under the same conditions.

The ability of the *phaZ *mutant strain to withstand long-term carbon starvation was tested, relative to both Rm1021 and Rm11105, by incubation for 4 weeks in M9 liquid medium with no added carbon source. Cells were grown to late-log in YMB and washed twice in M9. A 1:50 dilution was used to inoculate 75 ml of M9 salts. Starting cfu/ml was determined immediately following inoculation by serial dilution of a 1 ml aliquot. Starting cultures typically contained approximately 2 × 10^5 ^cfu/ml. These starting values were each given a relative value of 1. 1 ml samples were removed at 7 day intervals and serial dilutions were used to determine cfu/ml. Values presented are the averages of 3 independent cultures. The data in Figure. 1 show that the ability to synthesize and/or break down PHB has a significant impact on long-term survival in the absence of an exogenous carbon source. The wild-type strain Rm1021 is capable of increasing cell density during the early stages of starvation, presumably by degrading readily mobilizable intracellular carbon stores, a pattern which is not seen in either the *phaZ *or *phaC *mutants.

### PHB accumulation

To assess the effect of the *phaZ *lesion on PHB content in Rm11430, total PHB accumulation of stationary-phase cells was measured and compared to the wild-type strain Rm1021. Cells were grown to stationary phase in either YMB and the accumulated PHB was measured as a total cellular dry weight (% w/w). These data are shown in Table 2 and represent the average from three samples. Rm11430 demonstrates significantly increased PHB accumulation relative to Rm1021 suggesting that, while synthesis of PHB is not impaired, the lesion in *phaZ *inhibits degradation of PHB. The PHB accumulation phenotype of Rm11430 is complemented by pMA157, demonstrating a clear relationship between the presence of *phaZ *and PHB accumulation.

**Table 2:** PHB accumulation during free-living growth in Yeast-Mannitol Medium

Strain	Relevant Characteristics	PHB Accumulation % cell dry mass
Rm1021	wild-type	18.9
Rm11105	*phaC*::Tn*5*	0.240
Rm11430	*phaZ*ΩSmSp	28.6
Rm11430 pMA157	*phaZ*ΩSmSp pRK7813 *phaZ*	7.39

### Effect on expression of succinoglycan synthesis genes

The product of the *exoF *gene is involved in the transfer of the first sugar, galactose, to the lipid carrier, upon which the subunits of succinoglycan are assembled [25]. pD82*exoF*::Tn*phoA *was constructed by homologous recombination between *exoF *carried on pD82 [26] and the chromosomal *exoF*::Tn*phoA *fusion of strain Rm8369 [27]. The resultant plasmid was used to measure the transcriptional activity of *exoF *in different *S. meliloti *PHB mutant backgrounds when grown under different culture conditions. A Student's t-test was used to analyze the data and determine statistical significance of the observed differences. The results presented in Table 3 represent the mean of three independent samples. When analyzed using a two-tailed Student's t-test, the 1.1-fold increase in *exoF *expression exhibited by YMB-grown Rm11430 is statistically significant. Furthermore, the non-mucoid mutants Rm11105 and Rm11107 exhibit a reduction in *exoF *expression. This is consistent with the observation that colonies formed by Rm11430 appear larger and more mucoid on YMA than Rm11105 or Rm11107 (Table 1).

**Table 3:** *exoF::phoA *Alkaline Phosphatase Assay

Strain	Relevant Characteristics	Activity (U)	Std Error
Rm1021	wild-type	14.1	0.331
Rm11105	*phaC*::Tn*5*	9.68^*a*^	0.264
Rm11347	*phaB*O	6.23^*a*^	0.223
Rm11107	*bdhA*::Tn*5*	16.1	0.714
Rm11430	*phaZ *OSmSp	15.7^*a*^	0.296

^*a *^These differences are statistically significant from the value recorded for Rm1021, when analysed using a two-tailed Student's t-test

### Symbiotic phenotype of Rm11430 and bacteroid PHB accumulation

**Figure 2:**
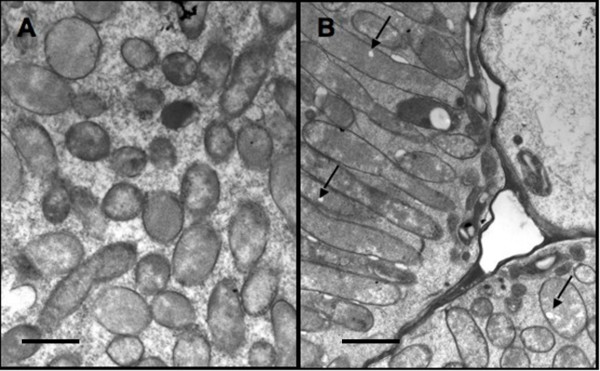
**Bacteroids of Rm1021 (A) and Rm11430 (B). Electron-transparent PHB granules are clearly visible in bacteroids of Rm11430**. PHB granules in the cytoplasm of the Rm11430 bacteroids are indicated in panel B. These granules are notably absent in the bacteroids of Rm1021 shown in panel A. Scale bar: 2 *μ*m.

Unlike bacteroids of determinate nodules, bacteroids of *S. meliloti *do not accumulate PHB during symbiosis (reviewed in [4]). Interestingly, a mutant of *R. leguminosarum *unable to cycle amino acids between the bacteroid and plants, showed apparent accumulation of PHB in the bacteroid within pea indeterminate nodules [11]. This suggests that the pathway for PHB metabolism can function within bacteroids of indeterminate nodules; however accumulation of PHB only occurs under extreme circumstances for example, when carbon is in excess and bacteroid metabolism is limited by the availability of a key nutrient. To confirm that *S. meliloti *bacteroids are capable of PHB synthesis and accumulation, alfalfa nodules induced by Rm11430 were prepared, sectioned and analyzed by TEM. Figure 2(a) clearly shows that bacteroids of Rm11430 accumulate PHB during symbiosis, with numerous, electron-transparent, PHB granules visible within the cytoplasm of the bacteroids when viewed by TEM. This is in contrast to bacteroids of Rm1021, shown in Figure 2(b), which demonstrate a notable absence of PHB.

Symbiotic assays with the host plant alfalfa revealed no significant difference between the *phaZ *mutant Rm11430 and the wild-type strain Rm1021. Plants inoculated with Rm11430 had an average shoot dry mass (SDM) of 10.56 mg compared to 10.80 mg for plants inoculated with Rm1021, both of which were significantly different to the uninoculated controls, which had an average SDM of 4.16 mg. This is interesting since it suggests that PHB accumulation, as confirmed in Figure 2, does not occur at the expense of symbiotic effectiveness.

### Competitiveness for nodule occupancy of Rm11430

The ability of *S. meliloti *Rm11430 to compete for nodule occupancy was assayed by co-inoculating alfalfa plants with different strain combinations. Table 4 shows that, when co-inoculated in approximately equal ratios with the wild-type strain, Rm11430 demonstrated no discernible difference in competitiveness relative to Rm1021. The percentage of Rm11430 in the original inoculum was similar to the percentage of nodules that it occupied. In agreement with previous studies [28], both Rm11105 (*phaC*) and Rm11107 (*bdhA*) demonstrated significantly reduced competitiveness relative to wild-type. Table 4 also shows that both Rm11105 and Rm11107 demonstrate reduced competitiveness relative to Rm11430, with the *phaC *phenotype being more pronounced than the *bdhA *phenotype.

**Table 4:** Nodulation competitiveness of the *S. meliloti *wild-type strain and *bdhA*, *phaC *and *phaZ *mutants co-inoculated in the described ratios on *M. sativa *plants

Strain (%) in inoculum	No. nodules tested	Nodule occupancy (%)		
		Strain 1	Strain 2	Both
Rm11430 (60) + Rm1021 (40)	18.0	61.1	22.2	16.7
Rm11430 (91) + Rm1021 (9)	15.0	93.3	6.7	0
Rm11430 (54) + Rm11105 (46)	16.0	100	0	0
Rm11105 (59) + Rm1021 (41)	15.0	6.70	93.3	0
Rm11105 (88) + Rm1021 (12)	20.0	5.00	75.0	20.0
Rm11430 (51) + Rm11107 (49)	20.0	65.0	35.0	0
Rm11107 (49) + Rm1021 (51)	14.0	21.4	78.6	0
Rm11107 (77) + Rm1021 (23)	15.0	86.7	0	13.3
Rm11107 (44) + Rm11144 (56)	19.0	94.7	0	5.30

The role of EPS in the establishment of nitrogen-fixing symbioses between *S. meliloti *and *M. sativa *has long been acknowledged [29], but the precise mechanism of interaction remains elusive. Mutants unable to synthesize EPS are characteristically Fix^-^. The observation that *phaC *and *phaB *mutants of *S. meliloti *are still able to establish successful symbioses [24] suggests that synthesis of succinoglycan in these mutants, albeit at a reduced level, is still sufficient to facilitate nodulation. This is consistent with previous reports which suggest that the production of small amounts of low-molecular-weight (LMW) EPS is sufficient to establish a successful symbiosis [29]. Indeed, it is conceivable that the competition defect observed in *phaC *mutants of *S. meliloti *may be due to extremely low levels of succinoglycan production. The *phaC *mutant may produce sufficient succinoglycan to establish an effective symbiosis but, assuming that the succinoglycan itself is playing a role in signalling during early nodulation, not enough to allow it to compete with strains producing higher levels of the EPS. Interestingly, the *phaZ *mutant demonstrates wild-type competitiveness and is able to out-compete both the *phaC *and *bdhA *mutants for nodulation. It is conceivable that another metabolic pathway that is dependent on D-3-HB metabolism may play a role in nodulation competitiveness. It is noteworthy that, although it has higher succinoglycan production than Rm1021, the *phaZ *mutant was not more competitive than the wild-type strain. While it is tempting to speculate that there may be a critical level of succinoglycan, above which, further gains in competitiveness are not seen, further information regarding the synthesis of succinoglycan during the infection process is still needed. Studies are currently underway in our lab to investigate this possibility further.

It is conceivable that, when PHB synthesis is inhibited, intermediates required for succinoglycan are not synthesized efficiently. It is also possible that, in the absence of a functional PHB synthesis pathway, enzymes required for succinoglycan may be inhibited or down-regulated. Furthermore, it has been suggested that acetyl phosphate may provide a regulatory link between PHB and succinoglycan synthesis [30]. Studies in the thermophilic cyanobacterium *Synechococcus *sp. strain MA19, have shown that acetyl phosphate is involved in the post-translational regulation of PHB synthase *in vitro*, and that this regulation is concentration-dependent [30]. As well, that study revealed that the enzyme phosphotransacetylase, which converts acetyl-CoA to acetyl phosphate, is only active under PHB-accumulating conditions [30]. In *E. coli*, acetyl phosphate is known to act as a global signal which acts through two-component regulatory signals [31], perhaps by direct phosphorylation of the response regulator [32] itself. Furthermore, the ChvI protein, of the *S. meliloti *ExoS-ChvI two-component regulatory system, is able to autophosphorylate in the presence of acetyl phosphate *in vitro *[33]. Since PHB synthesis mutants may excrete excess acetyl-CoA, levels of acetyl phosphate will likely be low under these conditions. Therefore, intracellular levels of acetyl phosphate may be an important factor in the ExoS-ChvI-dependent regulation of succinoglycan synthesis.

## Conclusions

Previous studies have demonstrated that the ability of certain bacteria to synthesize, accumulate and metabolize intracellular PHB stores is important in enhancing their capacity to survive unfavourable growth conditions [34-37]. Rhizobia in the soil environment must contend with varying nutrient conditions, from the carbon-deficient bulk soil, to the carbon-rich rhizosphere [33]. The ability to accumulate and utilize carbon stores would be highly advantageous, allowing rhizobia to cope with fluctuating carbon conditions, and thus, make them more competitive against other bacterial populations [38]. Previous studies have shown that mutant strains of *S. meliloti *unable to synthesize (*phaC*) or degrade (*bdhA*) PHB show a significant reduction in competitiveness for nodule occupancy [28,39], with mutants that are unable to synthesize PHB exhibiting a much greater loss in competitiveness than those unable to degrade PHB [28], as we have confirmed here.

This is the first study in which the competitiveness of an *S. meliloti phaZ *mutant has been investigated. It was expected, based upon the phenotype of the *bdhA *mutant [28], that the *phaZ *mutant would exhibit reduced nodulation competitiveness. Interestingly, the *phaZ *mutant was as competitive as wild-type in co-inoculation experiments, and consistently out-competed both *phaC *and *bdhA *mutants (Table 4). Studies in *Azotobacter vinelandii *have demonstrated a role for PHB in protection of the cell against environmental stresses including pH, oxidative stress and UV damage [40]. It is conceivable that the enhanced competitiveness of the *phaZ *mutant, relative to the *phaC *and *bdhA *mutants, is due to an enhanced ability to tolerate the conditions encountered in the soil and rhizosphere as a result of the increased cytoplasmic PHB concentration.

Interestingly, the *phaZ *mutant shows a similar reduction in long-term survival during starvation to the *phaC *mutant (Figure 1). This suggests that the inability to degrade PHB is just as detrimental to the cells as the inability to accumulate it. This also confirms that PHB degradation does play a significant role in fuelling cellular metabolism under adverse conditions, and that glycogen synthesis and degradation is not able to replace the function of PHB metabolism under these conditions.

Previous studies have shown that *S. meliloti *mutants defective in PHB synthesis also exhibit a significant reduction in succinoglycan production under conditions favouring both succinoglycan and PHB production [41], suggesting that these pathways share a common regulatory factor. *S. meliloti phaB *and *phaC *mutants exhibit non-mucoid colony morphology on carbon-rich media, while *bdhA *mutants show a mucoid colony morphology. This study further augments these observations by showing that a *phaZ *mutant is not only mucoid, but has up-regulated exopolysaccharide production relative to the wild-type strain.

Bacteroids of determinate nodules, in contrast to those found in indeterminate nodules, can accumulate up to 50% of their cellular dry mass as PHB (reviewed in [4]). The synthesis of PHB during symbiosis however, presumably occurs at the expense of symbiotic nitrogen fixation; a theory that is corroborated by the observation that a *phaC *mutant of *R. etli *demonstrates higher levels of nitrogenase activity relative to wild-type [42]. Bacteroids of indeterminate nodules do not accumulate PHB during symbiosis. It has been suggested [42] that this may be one of the reasons why the *S. meliloti*-alfalfa symbiosis is more effective than that of *B. japonicum*-soybean or *R. etli*-bean [43]. Interestingly the data presented in this paper suggest that forced accumulation of PHB by *S. meliloti *during symbiosis does not appear to have a negative effect on plant yield, suggesting that PHB synthesis during symbiosis is not the only determinant of symbiotic performance.

## Methods

### Bacterial strains, plasmids, growth media and conditions

All bacterial strains and plasmids used are listed in Table 5. Culture methods using Tryptone Yeast (TY), Luria Broth (LB), Yeast Mannitol Broth (YMB), Yeast Mannitol Agar (YMA), and Modified M9 medium supplemented with defined carbon sources, and antibiotic concentrations were carried out as described previously [23,44].

**Table 5:** Bacterial Strains, Plasmids and Phage

Strain or Plasmid	Relevant Characteristics	Reference
***S. meliloti***		
Rm5000	SU47 *rif5*	[22]
Rm1021	SU47 str-21, Sm^*R*^	[50]
Rm11105	Rm1021 *phaC *1::Tn*5*	[23]
Rm11107	Rm1021 *bdhA*1::Tn*5*	[23]
Rm11144	Rm1021 *phaC*1::Tn*5 *-233	[23]
Rm11347	Rm1021 *phaB*::ΩSmSp	[24]
Rm11417	Rm5000 *phaZ*::ΩSmSp	This work
Rm11430	Rm1021 *phaZ*::ΩSmSp	This work
Rm8369	Rm8002 *exoF*369::Tn*phoA*	[27]

***E. coli***		
DH5*α*	F' *endA1 hsdR17 *(r_*K*_m_+_) *supE44 *thi^-1 ^*recA1 gyrA *Nal^*R *^*relA1 *Δ(*lacIZYA-argF) U169 deoR *(*ϕ*80*dlac *Δ(*lacZ*)M15)	[51]
MT607	*pro-82 thi-1 hsdR17 supE44 recA56*	[52]
MT616	MT607 pRK600	[52]

**Plasmids**		
pK19*mobsacB*	Suicide vector Km^*R*^	[53]
pGEMTEasy	Cloning vector for PCR-generated DNA fragments, Amp^*R*^	Promega
pAZ101	pGEMTeasy carrying 835 bp fragment of SMc02770	This work
pAZ102	pAZ101 *phaZ*::OSmSp	This work
pAZ103	pK19*mobsacB phaZ*::ΩSmSp	This work
pRK7813	RK2 derivative carrying pUC9 polylinker. Tc^*R*^	[54]
pMA157	pRK7813 SMc02770	This work
pD82	pLAFR1 cosmid clone from Rm1021 library carrying *exoF *and neighbouring genes	[26]
pD82*exoF*::Tn*phoA*	pD82 *exoF*::Tn*phoA*	This work

**Phage**		
*ϕ*M12	*S. meliloti *transducing phage	[22]

### Genetics and molecular biology techniques

Bacterial conjugations, *ϕ*M12 transductions and homogenotizations were carried out as described previously [22]. DNA manipulations were performed using standard techniques [45]. DNA probes for Southern blot analyses were labelled with digoxygenin (DIG) using the DIG High-Prime Kit (Roche Diagnostics Canada) according to manufacturer's instructions. Southern blots were performed using standard techniques [45]. PCR was carried out by standard techniques [45] using KOD Polymerase (Novagen, Canada).

### Construction of exoF::TnphoA fusion

To generate plasmid-borne *exoF*::Tn*phoA *fusions, plasmid pD82, a cosmid clone carrying the *S. meliloti exoF *gene and surrounding region of the genome [26], was introduced into the *S. meliloti exoF*::Tn*phoA *fusion strain Rm8369 [27]. This construct was subsequently transferred into *E. coli *strain MT607, by triparental conjugation using *E. coli *strain MT616 as the mobilizer. Transconjugants were selected on LB KmTc, and the nature of the fusion was confirmed by testing for inability to confer YMA mucoidy on the *exoF*::Tn*phoA *mutant Rm7055. The resulting construct was named pD82 *exoF*::Tn*phoA*.

### Biochemical assays

Alkaline phosphatase activity of *exoF*::Tn*phoA *fusions in *S. meliloti *strains was measured according to the method of Brinkmann and Beckwith [46]. Cells were grown to an OD_600 _of 0.7. 1 ml of culture was washed twice in 1 M Tris-HCl (pH 8.0), and resuspended in 1 ml 1 M Tris-HCl (pH 8.0). The OD_600 _of this cell suspension was then measured. Following a 10 min equilibration period at 37°C, 50 *μ*l of 4 mg/ml *p*-nitrophenyl phosphate (NPP) was added to start the reaction. The reaction was allowed to continue for 11 min at 37°C before being stopped by the addition of 50 *μ*l of 1 M K_2_HPO_4_. The cells were pelleted and 50 *μ*l of the supernatant was diluted in 450 *μ*l of 1 M Tris-HCl (pH 8.0) and OD_420 _was measured. Units (U) of alkaline phosphatase activity were calculated using the formula:(1)

Assuming a molar coefficient of 16,000 for p-nitrophenyl phosphate, 1 U is equal to 0.062 nmol of NPP hydrolyzed per min at a cell OD_600 _of 1. Therefore:(2)

For PHB assays, 50 ml cultures were grown at 30°C to stationary phase in YMB. Cells were harvested and washed in 0.85% NaCl solution before resuspension in 50 ml 0.85% NaCl. PHB was extracted from a 2 ml fraction of this suspension and the remaining 48 ml was used for cell dry weight determination by incubation of the pellet at 60°C until the pellet was dry and no further loss in mass was recorded. PHB content was measured by the method of Law and Slepecky [47] and expressed as a percentage of total cell dry weight. All glassware was washed in hot chloroform and rinsed in ethanol before use, to eliminate plasticizers. A standard curve was constructed by dissolving known quantities of PHB (Sigma) in hot chloroform to a final volume of 1 ml. The chloroform was allowed to evaporate before addition of 10 ml of H_2_SO_4 _and PHB was processed as described elsewhere [47].

### Carbon starvation assay

Saturated TY cultures were washed twice to remove traces of nutrients, and were subcultured 1:50 into carbon-free M9 medium. These cultures were incubated at 30°C, shaking at 180 rpm. Viable cell counts were monitored at weekly intervals by plating on TY agar. Samples at t = 0 were each given a relative value of 1, and all subsequent samples are compared to this starting value. Values recorded are the means from triplicate cultures.

### Nodulation studies

*Medicago sativa *cv. *Iroquois *seeds were surface-sterilized in 95% ethanol for 2 min followed by 15 min in 0.5% sodium hypochlorite. After several washes in sterile dH2O, seeds were germinated in the dark on sterile water agar plates at room temperature for approximately 36 hours. Seedlings were transferred to modified Leonard assemblies containing sterilized vermiculite soaked in Jensen's N-free plant nutrient solution [48]. Five seedlings were planted in each jar and inoculated with 5 ml of 1:50 dilution of saturated TY culture. The assemblies were placed in a growth chamber (Conviron CMP3244, Model # EF7, Controlled Environments Ltd., Winnipeg) with 16 h, 25°C day/8 h, 20°C night and light intensity of 300 *μ*moles m^-2^s^-1^. For shoot dry weight determination, plants were harvested approximately 5 weeks post-inoculation and the shoots separated from the roots. The shoots were transferred to brown paper bags and incubated at 60°C until no further loss in mass was recorded. Shoot dry weight is expressed as mg^-1 ^plant^-1^. Nodule occupancy competitiveness was assayed in modified Leonard assemblies as described above. Inoculants consisted of wild-type and mutant cultures mixed in 1:1 and 1:9 ratios, or mutant cultures mixed in a 1:1 ratio. Plants were harvested four weeks post-inoculation and nodules were collected. Nodules were surface-sterilized with 1% sodium hypochlorite (15 min), washed twice with LB, and then squashed in a few drops of TY containing 0.3 M sucrose. The resultant suspension was streaked on TY. Four colonies isolated from each nodule were screened for the appropriate antibiotic-resistance marker. The bacterial population within each nodule was thus scored as either consisting of one strain or a mixture of two strains.

### Electron microscopy

*M. sativa *plants were harvested 28-30 days post-infection. Roots were washed to remove traces of vermiculite, and the nodules were transferred into primary fixative (4% formaldehyde, 1% glutaraldehyde in 80 mM HEPES pH 7.0) and cut into small pieces. The samples were subjected to 4 cycles of vacuum infiltration (2 mins per cycle) and were left overnight at 4°C. Following infiltration, the nodules were washed thoroughly in sterile water, and stained for 4 hours in 1% OsO_4_. The nodules were washed again in water and dehydrated through a gradient of acetone. The nodules were embedded in epon araldite resin and transferred to BEEM capsules for 48 hours at 60°C. Ultrathin sections were cut using a Reichert Ultracut E microtome, and were stained with uranyl acetate and lead citrate using standard techniques [49]. Samples were analyzed in a Philips CM10 transmission electron microscope at an accelerating voltage of 60 kV.

## Authors' contributions

AZ generated the *phaZ *mutant. MAT performed the cloning reactions, carbon starvation assays, symbiosis assays, electron microscopy, supervised KNL and drafted the manuscript. KNL conducted carbon starvation assays. TCC constructed the pD82*exoF*::Tn*phoA *vector. DC conducted the alkaline phosphatase assays and plant competition experiments. TCC and SRDC participated in experimental design and data analysis. All authors have read and approved the final manuscript.
